# Acute and Chronic Nicotine Exposures Differentially Affect Central Serotonin 2A Receptor Function: Focus on the Lateral Habenula

**DOI:** 10.3390/ijms21051873

**Published:** 2020-03-09

**Authors:** Cristiano Bombardi, Francis Delicata, Claudio Tagliavia, Massimo Pierucci, Gabriele Deidda, Maurizio Casarrubea, Philippe De Deurwaerdère, Giuseppe Di Giovanni

**Affiliations:** 1 Dipartimento di Scienze Mediche Veterinarie, Università di Bologna, Bologna, Via Tolara di Sopra, 50, 40064 Ozzano dell’Emilia (BO), Italy; cristiano.bombardi@unibo.it (C.B.); claudio.tagliavia2@unibo.it (C.T.); 2 Department of Physiology and Biochemistry, Laboratory of Neurophysiology, Faculty of Medicine and Surgery, University of Malta, MSD2080 Msida, Malta; francis.delicata.06@um.edu.mt (F.D.); Massimo.pierucci@um.edu.mt (M.P.); gabriele.deidda@um.edu.mt (G.D.); 3 Department of Biomedicine, Neuroscience and Advances Diagnosis (BIND), Human Physiology Section “Giuseppe Pagano”, Laboratory of Behavioral Physiology, University of Palermo, Viale delle Scienze n. 13, 90128 Palermo, Italy; maurizio.casarrubea@unipa.it; 4 Centre National de la Recherche Scientifique (Unité Mixte de Recherche 5287), 146 rue Léo Saignat, B.P.281, F-33000 Bordeaux Cedex, France; philippe.de-deurwaerdere@u-bordeaux.fr; 5 School of Biosciences, Cardiff University, Cardiff CF10 3AX, UK

**Keywords:** 5-HT_2C_R, nucleus accumbens, dorsal raphe nucleus, addiction, depression, medial prefrontal cortex, dentate gyrus, striatum, ventral tegmental area, substantia nigra pars compacta

## Abstract

Nicotine addiction is a serious public health problem causing millions of deaths worldwide. Serotonin (5-hydroxytryptamine; 5-HT) is involved in central nervous system (CNS) nicotine effects, and it has been suggested as a promising pharmacological target for smoking cessation. In this regard, what is particularly interesting are the 5-HT_2A_ receptors (5-HT_2A_Rs) and the lateral habenula (LHb), a central area in nicotine addiction that we showed to be under a strong 5-HT_2A_R-modulation. Single-cell extracellular recording of LHb neurons was used to study the 5-HT_2A_R function by intravenously administrating the potent agonist TCB-2. Acute nicotine (2 mg/kg, intraperitoneal, i.p.) and chronic nicotine (6 mg/kg/day for 14 days) differently affected both the 5-HT_2A_R-immuno reactive (IR) neuron number and the 5-HT_2A_R immunostaining area in the different brain areas studied. After acute nicotine, TCB-2 cumulative doses (5–640 µg/kg, intravenous, i.v.) bidirectionally affected the activity of 74% of LHb recorded neurons. After chronic nicotine treatment, TCB-2 was only capable of decreasing the LHb firing rate. The expression of 5-HT_2A_R under acute and chronic nicotine exposure was studied in the LHb and in other brain areas involved in nicotine effects in rats by using immunohistochemistry. These data reveal that acute and chronic nicotine differentially affect the 5-HT_2A_R function in different brain areas and this might be relevant in nicotine addiction and its treatment.

## 1. Introduction

Cigarette smoking is one of the most common addictions that is decreasing in Western societies, but dramatically increasing in third world countries [1]. Cigarette smoke is the primary avoidable cause of disability and death from several pathologies related to different organs, mainly respiratory and cardiovascular systems [2,3,4]. Considering that about 8 million people die every year as a consequence of nicotine addiction, smoking is among the most significant threat to world health. Nicotine, the primary molecule contained in tobacco leaves, and responsible in the development of addiction [5,6,7], is an exogenous agonist of the nicotinic cholinergic receptors (nAChRs). The addictive properties of this alkaloid depends primarily on the activation of the dopamine (DA) mesolimbic DA system [8,9], although, nicotine is known to affect many other neurotransmitters in the central nervous system (CNS), such as glutamate (GLU) [10], nitric oxide [11], gamma-aminobutyric acid (GABA) [12], and serotonin (5-hydroxytryptamine; 5-HT) [4,13]. Moreover, 5-HT and its receptors may be a key to tobacco addiction. Extensive and compelling animal and human data have recently revealed an essential interaction between nicotine and 5-HT_2C_ receptors (5-HT_2C_Rs) that might open new therapeutic avenues to smoking cessation [14,15,16,17]. On the other hand, the 5-HT_2A_Rs have been less investigated, although it is known that the 5-HT_2A_R exerts a distinct role compared to 5-HT_2C_Rs concerning the regulation of midbrain DA neurons [18,19], ultimately leading to an opposite influence of the two 5-HTRs in nicotine addiction. Indeed, their blockade is able to counteract nicotine self-administration, reinstatement, sensitization, conditioned hyperactivity, and withdrawal behaviors [17,20,21]. Even if 5-HT_2A_Rs are expressed in brain areas involved in nicotine addiction [22,23], the effect of nicotine on their expression has not yet been thoroughly investigated. The only evidence comes from an autoradiographic study showing that acute nicotine increased [^3^H] ketanserin binding to 5-HT_2A_Rs in the claustrum and ventral tegmental area (VTA) while short chronic (five days) nicotine treatment increased them also in the medial prefrontal cortex (mPFC), dorsal striatum (ST), shell of the nucleus accumbens (NAc)—but not in the claustrum [24].

Of particular interest in studying drug addiction is the lateral habenula (LHb), a small epithalamic structure that conveys negative motivational signals [25], which might be an essential hub in nicotine/5-HT_2A_R interaction. Indeed, the LHb can be directly activated by nicotine [26,27,28], and its lesion prevented nicotine-induce anxiety [29]. The LHb is heavily modulated by the serotonergic system and expresses several 5-HTR subtypes [30,31]. Of note, the 5-HT_2A_R seems to be one of the most critical 5-HTRs in modulating LHb neuronal activity also in comparison to 5-HT_2C_Rs. Nevertheless, the effect of acute and chronic nicotine on anatomical distribution of the 5-HT_2A_R protein in LHb and many other brain areas related to nicotine addiction remains obscure. Moreover, it is not known if nicotine treatment can change the 5-HT_2A_R modulation of the electrical activity of the target brain areas.

In the present study, to fill this gap, we studied the effect of the intraperitoneal (i.p.) administration of acute and chronic nicotine (2 mg/kg and 6 mg/kg/day for 14 days, respectively) on the 5-HT_2A_R protein expression in the LHb. To study whether plastic functional changes of the 5-HT_2A_R signaling occur in the LHb, we have studied the effect of intravenous (i.v.) administration of the 5-HT_2A_R agonist TCB-2 [32] on the neuronal activity of LHb neurons using extracellular single-cell recordings in vivo in acute and nicotine-treated rats. We found that acute and chronic nicotine treatments did not change the number of LHb 5-HT_2A_R-immuno reactive (IR) neurons, while only acute nicotine increased their neuropil expression. On the other hand, TCB-2 (5–640 µg/kg, i.p.) in acute nicotine-treated rats induced both excitations and inhibitions of LHb neural activity while in chronic conditions, only inhibitions were observed.

We also studied the 5-HT_2A_R expression in other areas involved in nicotine addiction such as the mPFC, hippocampal dentate gyrus (DG), the NAc, the ST, the VTA, the substantia nigra pars compacta (SNc), and the dorsal raphe nucleus (DRN) in rats. We observed only in the DG an increase in the number of 5-HT_2A_R-IR neurons upon acute nicotine treatment, while chronic treatment normalized their number in comparison to drug naïve values. The neuropil 5-HT_2A_R expression was decreased in mPFC and increased in the VTA in acute nicotine-treated rats. Chronic nicotine-induced normalization in mPFC, DG, increased in NAc, and decreased in the ST. The present data indicate that acute and chronic nicotine exposure induces differential 5-HT_2A_R changes within the brain areas studied that might be responsible for the differences in the LHb neuronal response to TCB-2 administration in the acute and chronic conditions.

## 2. Results

### 2.1. Effect of Systemic Administration of Acute and Chronic Nicotine on 5-HT_2A_R Immunohistochemistry Expression in the LHb

**Figure 1:**
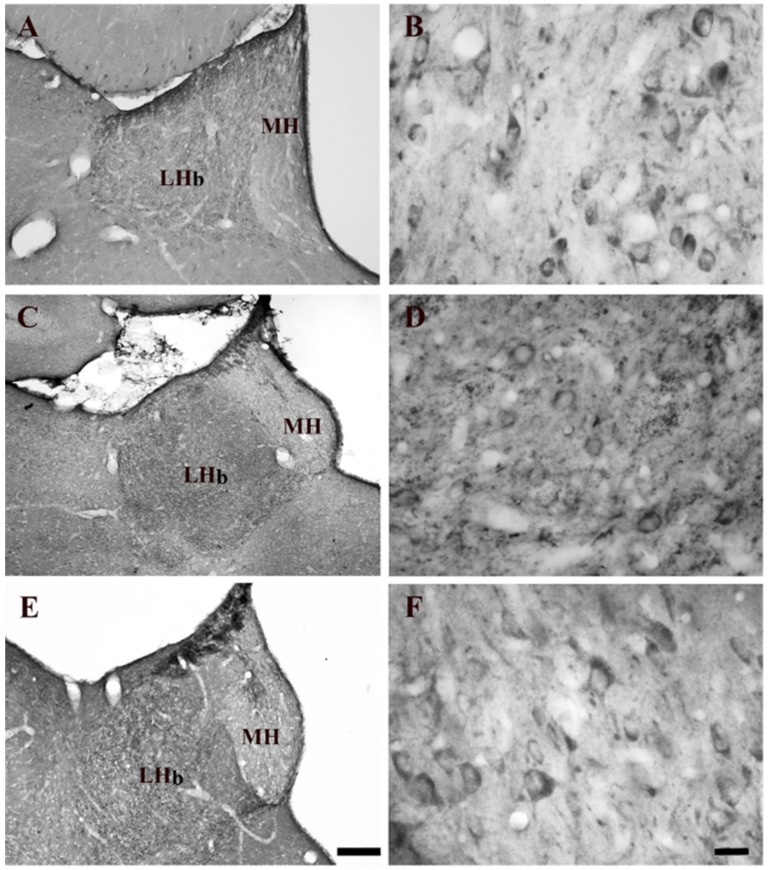
Brightfield photomicrographs of coronal sections showing the distribution of 5-HT_2A_ receptors (5-HT_2A_Rs) immunoreactivity in the lateral habenula (LHb) of nicotine-naïve (**A**,**B**), acute nicotine (**C**,**D**), and chronic nicotine (**E**,**F**). Note the high 5-HT_2A_R immunoreactivity in the LHb of acute nicotine rats (**C**). See text and Table 1 explanations. Abbreviations: LHb, lateral habenula; MH, medial habenula. Scale bar = 200 µm in E (applies to **A**,**C**,**E**) and 20 µm in F (applies to **B**,**D**,**F**).

The LHb of drug naïve rats had a moderate intensity of 5-HT_2A_R immunostaining (Figure 1A,B). The density of 5-HT_2A_R immunostained somata was similar among the different experimental groups (naïve nicotine, acute and chronic nicotine) (Figure 1A–F). However, the intensity of the neuropil staining was low (Figure 1A,B). The intensity of 5-HT_2A_R-immunostaining was evident in the LHb of acute nicotine rats. Statistical analysis did not show any significant differences comparing the neuronal densities observed in the different groups. 

**Figure 2:**
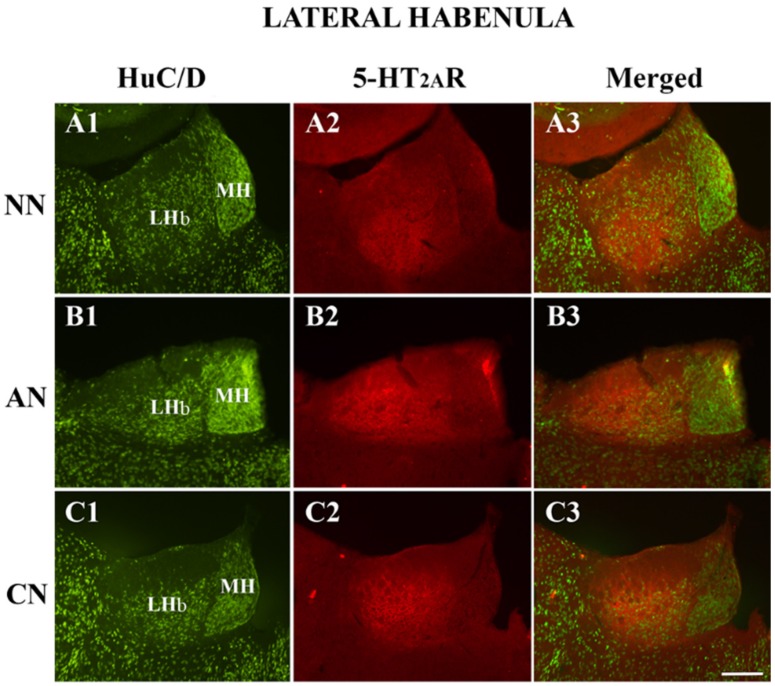
Colocalization of the HuC/D (pan-neuronal marker) with 5-HT_2A_ receptor (5-HT_2A_R) in the lateral habenula of naive nicotine (A1–A3), acute nicotine (B1–B3) and chronic nicotine (C1–C3) rats. Double immunofluorescence images showing HuC/D in green (left column pictures; A1, B1, C1), 5-HT_2A_R in red (middle column pictures; A2, B2, C2), and colocalization of HuC/D with 5-HT_2A_R in yellow (right column merging pictures; A3, B3, C3). Note the high 5-HT_2A_R immunoreactivity in the LHb of acute nicotine rats. See text and Table 2 for explanations. Abbreviations: NN, naïve nicotine; AN, acute nicotine; CN, chronic nicotine; LHb, lateral habenula; MH, medial habenula. Scale bar = 200 μm in C3 (applies to A1–C3).

The double immunofluorescence analysis, performed in the LHb consisted of the colocalization of the 5-HT_2A_R with HuC/D (pan-neuronal marker). We did not observe any significant differences in the proportion of 5-HT_2A_R-IR, comparing the different groups (Figure 2; Table 1). The percentage of the image covered by 5-HT_2A_R immunoreactivity showed significant differences in the LHb (Table 1, acute nicotine > nicotine-naïve) confirming immunoperoxidase experiments.

### 2.2. 5-HT_2A_R Electrophysiology Experiments

#### 2.2.1. Electrophysiological Characteristics of Spontaneously Active the LHb Neurons

We recorded 70 spontaneously active neurons extracellularly in the LHb. The neurons fired at 11.9 ± 0.67 Hz with a waveform duration of 1.1 ± 0.01 ms. The LHb neurons recorded had a predominantly (82.9%) biphasic waveform and three distinct firing patterns; the majority were irregular (82.9%), while regular and bursty neurons contributed to less than 10%. The average coefficient of variation (CV) was 0.74 ± 0.03, and as expected was greatest in burst firing neurons and lowest in regular firing neurons (data not shown). 

#### 2.2.2. Effect of Systemic Administration of 5-HT_2A_R Agonist TCB-2 on the Firing Rate and Pattern of LHb Neurons of Acute and Chronic-Treated Rats

**Figure 3:**
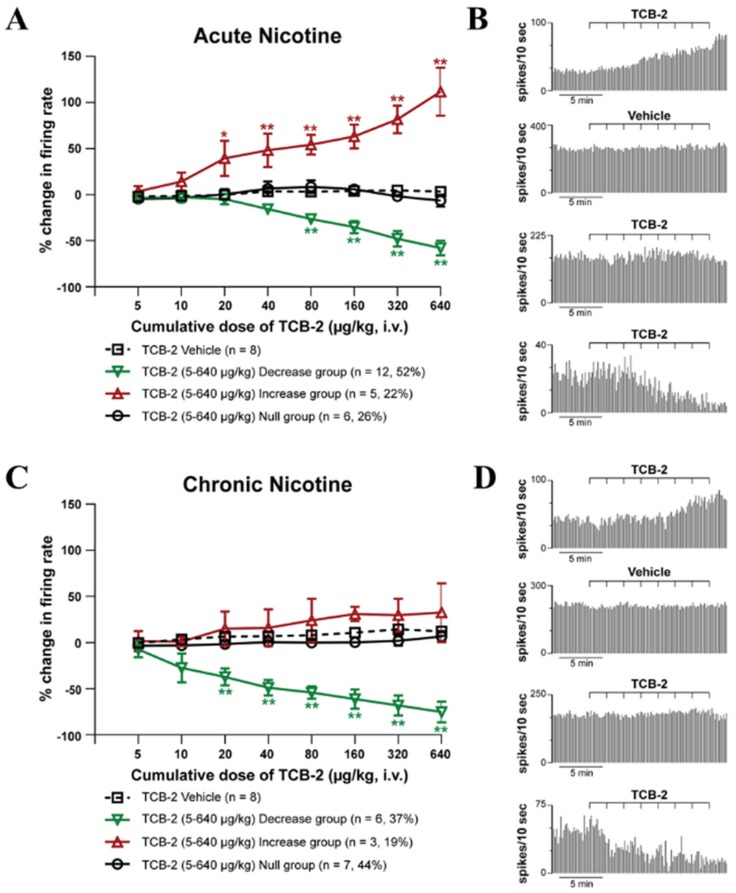
(**A**). The dose-response curve of TCB-2 (5–640 μg/kg, iv) 60 min after a single acute treatment of nicotine (2 mg/kg, i.p.). The data are shown as the mean % change in firing rate ± SEM. The majority of neurons (52%) responded with a decrease in firing rate, with peak effect at 640 μg/kg (−57.8 ± 7.9%). The remaining neurons responded with an increase in firing rate (22%, peak effect at 640 μg/kg, 111.7 ± 26.1%), or showed no change in firing rate (26%). One-way ANOVA with repeated-measures, followed by Tukey’s post hoc test, * *p* < 0.05 vs Vehicle, ** *p* < 0.005 vs Vehicle. (**B**) Typical rate histograms of single neuronal recordings that showed an increase in firing, a control neuron, no change in firing, and a decrease in firing (sequentially from top to bottom). (**C**) A dose-response curve of TCB-2 (5–640 μg/kg, i.v.) after chronic treatment of nicotine (6 mg/kg/day for 14 days). Almost an equal number of neurons responded with no change in firing rate (44%) or with a decrease in firing rate (37%, peak effect at 640 μg/kg, −75.0 ± 11.4%). The remaining neurons responded with a small, not significant increase in firing rate (19%, peak effect at 640 μg/kg, 32.7 ± 31.4%). (**D**). Typical rate histograms of single neuronal recordings that showed an increase in firing, a control neuron, no change in firing, and a decrease in firing (sequentially from top to bottom). One-way ANOVA with repeated-measures, followed by Tukey’s post hoc test, * *p* < 0.05 vs Vehicle, ** *p* < 0.005 vs. Vehicle.

ANOVA analysis shows that the administration of TCB-2 (5–640 μg/kg, iv) after 60 min from the acute nicotine-treatment (2 mg/kg; i.p.) induced a significant increase and decrease of the LHb neuronal firing rate (Figure 3A,B). 

After chronic treatment of nicotine (6 mg/kg/day for 14 days), TCB-2 (5–640 μg/kg, i.v.) induced almost in an equal number of neurons either no change in firing rate (*n* = 7; 44%) or a decrease in firing rate (*n* = 6; 37%) with a peak effect at 640 μg/kg of −75.0 ± 11.4% (*p* < 0.005; post-hoc Tukey test) when compared to the control group. The remaining neurons responded with no significant increase in firing rate (*n* = 3; 19%, peak effect at 640 μg/kg, 32.7 ± 31.4%, *p* = 0.585 post-hoc Tukey test Figure 3C,D). The chronic inhibitory group was not different from the acute inhibitory one at any of the doses tested (two-way ANOVA followed by Tukey’s test, *p* = 0.9). 

### 2.3. 5-HT_2A_R Colocalization Studies in the mPFC, DG, NAc, ST, VTA, SNc, and DRN 

**Figure 4:**
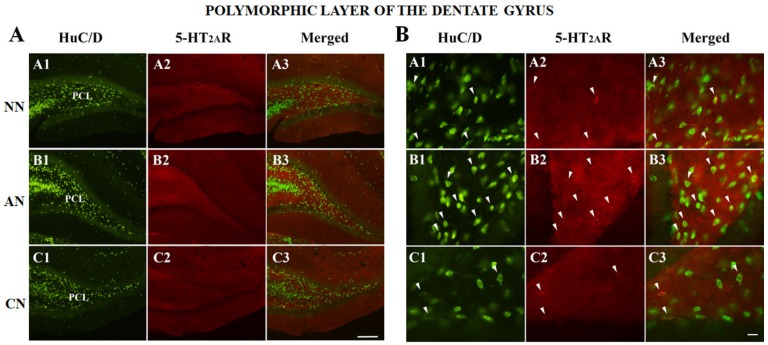
(**A**) Colocalization of the HuC/D with 5-HT_2A_ receptor (5-HT_2A_R) in the dentate gyrus (polymorphic cell layer) of naïve nicotine (NN) (A1–A3), acute nicotine (AN) (B1–B3) and chronic nicotine (CN) (C1–C3) rats. Double immunofluorescence images showing HuC/D in green (left column pictures; A1, B1 and C1), 5-HT_2A_R in red (middle column pictures; A2, B2 and C2), and colocalization of HuC/D with 5-HT_2A_R in yellow (right column merging pictures; A3, B3 and C3). Note the high 5-HT_2A_R immunoreactivity in the polymorphic cell layer of acute nicotine rats. See text and Table 2 for explanations. Abbreviations: PCL, polymorphic cell layer. Scale bar = 200 μm in C3 (applies to A1–C3). (**B**) Colocalization of the HuC/D with 5-HT_2A_R in the dentate gyrus (polymorphic cell layer) of nicotine-naïve (A1–A3), acute nicotine (B1–B3), and chronic nicotine (C1–C3) rats. Double immunofluorescence images showing HuC/D in green (left column pictures; A1, B1, and C1), 5-HT_2A_R in red middle column pictures; A2, B2, and C2), and colocalization of HuC/D with 5-HT_2A_R in yellow (right column merging pictures; A3, B3, and C3 arrowheads indicate double-labeled neurons). Note that in acute nicotine rats there are more d(ouble-labeled neurons than in nicotine-naïve and chronic nicotine rats. See text and Table 2 for explanations. Scale bar = 25 μm in C3 (applies to A1–C3).

**Figure 5:**
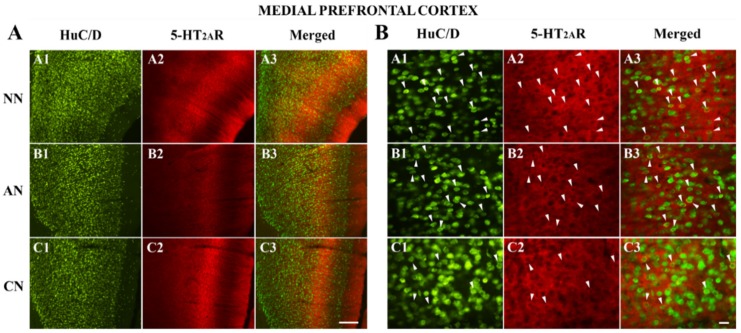
(**A**) Colocalization of the HuC/D with 5-HT_2A_ receptor (5-HT_2A_R) in the medial prefrontal cortex (mPFC, layers V and VI) of naïve nicotine (NN) (A1–A3), acute nicotine (AN) (B1–B3) and chronic nicotine (CN) (C1–C3) rats. Double immunofluorescence images showing HuC/D in green (left column pictures; A1, B1, and C1), 5-HT_2A_R in red (middle column pictures; A2, B2, and C2), and colocalization of HuC/D with 5-HT_2A_R in yellow (right column merging pictures; A3, B3, and C3). Note the high 5-HT_2A_R immunoreactivity in the mPFC of nicotine-naïve and chronic nicotine rats. See text and Table 2 for explanations. Scale bar = 200 μm in C3 (applies to A1–C3). (**B**) Colocalization of the HuC/D with 5-HT_2A_ receptor (5-HT_2A_R) in the mPFC (layers V and VI) of nicotine-naïve (A1–A3), acute nicotine (B1–B3), and chronic nicotine (C1–C3) rats. Double immunofluorescence images showing HuC/D in green (left column pictures; A1, B1, and C1), 5-HT_2A_R in red (middle column pictures; A2, B2, and C2), and colocalization of HuC/D with 5-HT_2A_R in yellow (right column merging pictures; A3, B3, and C3 arrowheads indicate double-labeled neurons). The proportion of 5-HT_2A_R-immunoreactive neurons to the total neurons does not show significant differences comparing the different groups. See text and Table 2 for explanations. Scale bar = 25 μm in C3 (applies to A1–C3).

**Figure 6:**
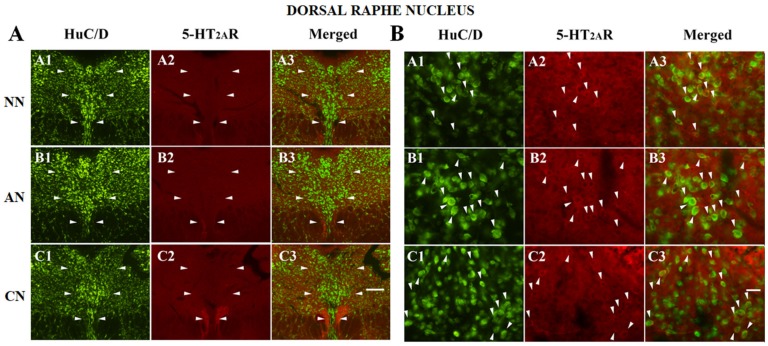
(**A**) Colocalization of the HuC/D with 5-HT_2A_ receptor (5-HT_2A_R) in the dorsal raphe nucleus (DRN, bordered by arrowheads) of naïve nicotine (NN) (A1–A3), acute nicotine (AN) (B1–B3), and chronic nicotine (CN) (C1–C3) rats. Double immunofluorescence images showing HuC/D in green (left column pictures; A1, B1, and C1), 5-HT_2A_R in red (middle column pictures; A2, B2, and C2), and colocalization of HuC/D with 5-HT_2A_R in yellow (right column merging pictures; A3, B3, and C3). The immunoreactivity for the 5-HT_2A_R is similar in the different groups. See text and Table 2 for explanations. Scale bar = 200 μm in C3 (applies to A1–C3). (**B**) Colocalization of the HuC/D with 5-HT_2A_R in the DRN of naive nicotine (A1–A3), acute nicotine (B1-B3), and chronic nicotine (C1-C3) rats. Double immunofluorescence images showing HuC/D in green (left column pictures; A1, B1, and C1), 5-HT_2A_R in red (middle column pictures; A2, B2, and C2), and colocalization of HuC/D with 5-HT_2A_R in yellow (right column merging pictures; A3, B3, and C3 arrowheads indicate double-labeled neurons). The proportion of 5-HT_2A_R-immunoreactive neurons to the total neurons does not show significant differences comparing the different groups. See text and Table 2 for explanations. Scale bar = 25 μm in C3 (applies to A1–C3).

The double immunofluorescence analysis, performed on mPFC, DG, ST, NAc, VTA, SNc, and DRN consisted of the colocalization of the 5-HT_2A_R with HuC/D (pan-neuronal marker) (Figure 4, Figure 5 and Figure 6 and Figures S1–S8). Except for the DG (polymorphic layer Figure 4), we did not observe any significant differences in the proportion of 5-HT_2A_R-IR, comparing the different groups (Figure 3, Figure 4, Figure 5 and Figure 6 and Figures S1–S8; Table 2). The proportion of 5-HT_2A_R-IR neurons in the polymorphic layer of the DG was significantly higher in acute nicotine rats than in nicotine-naïve and chronic nicotine rats (Figure 4; Table 2). In the polymorphic layers, the percentage of the image covered by 5-HT_2A_R immunoreactivity was significantly higher in acute nicotine rats than in chronic nicotine rats. In fact, in acute nicotine, there was an increase in the proportion of double-labeled cells (only for acute nicotine rats) and the neuropilar immunostaining (Figure 4; Table 2). The percentage of the image covered by 5-HT_2A_R immunoreactivity showed significant differences also in the mPFC (nicotine-naïve and chronic nicotine > acute nicotine), ST (acute nicotine > chronic nicotine), NAc (chronic nicotine > nicotine-naïve), and VTA (acute nicotine > nicotine-naïve) (Table 2). These differences were related to different neuropilar immunostaining. Accordingly, the proportion of 5-HT_2A_R-IR neurons did not show any significant difference in mPFC, ST, NAc, and VTA comparing the different groups (Table 2). 

## 3. Discussion

Here we reported for the first time that acute and chronic nicotine treatment affected the 5-HT_2A_R expression and electrophysiological functionality in the LHb. Indeed, acute nicotine (2 mg/kg, i.p.) increased the LHb 5-HT_2A_R neuropil expression while chronic nicotine treatment (6 mg/kg/day for 14 days, i.p.) decreased the neuropil IR to the nicotine-naïve levels. Of note, both acute and chronic nicotine treatments did not modify the number of LHb neurons expressing 5-HT_2A_Rs. Interestingly, chronic nicotine treatment changed the effect of peripheral administration of 5-HT_2A_R agonist TCB-2 [32,33,34], which was capable of producing only inhibitions of the LHb neurons, while both excitation and inhibition of the LHb neuronal activity were observed after acute nicotine treatment. 

A complicated pattern of effects by acute and nicotine exposure on these receptors in the areas known to be involved in the nicotine effects in the CNS was revealed. The increase of 5-HT_2A_R-IR neurons occurred only in the hippocampal DG in nicotine acute-treated rats. The most common effect of nicotine exposure was a change of the neuropil 5-HT_2A_R expression, either as a decrease in mPFC (acute nicotine) and the ST (chronic nicotine) or as an increase in the VTA (acute nicotine) and the NAc (chronic nicotine). Chronic nicotine treatment induced normalization of neuropil 5-HT_2A_R-IR in the mPFC and the DG.

Our data on the LHb further support the significant involvement of this small epithalamic area [28,29] and the 5-HT_2A_R system [17,35] and their interaction in nicotine effects in the brain and, in general, in drug dependency. Nicotine’s modulation is a complex phenomenon and, apart from the rewarding effect mediated by increasing DAergic transmission [7], this alkaloid modulates different neurotransmitter systems. Among them, nicotine affects 5-HT neurons and 5-HT-innervated brain areas [17,36]. For instance, the LHb receives a dense serotonergic innervation from the raphe nuclei [30], expresses several 5-HTR subtypes [30,31], the *HT2A* gene [37], and 5-HT_2A_R protein, both on neurons and glial cells [34]. Here, in line with our previous study, we found that only a small population of LHb neurons (9–8%) was 5-HT_2A_R-IR, independently from the nicotine treatment. Interestingly, we detected an increase of the area covered by 5-HT_2A_R-IR in acute nicotine-treated animals that was decreased by 14 days of nicotine treatment to drug-naïve animal levels. It is difficult to identify from our immunohistochemistry (IHC) evidence the precise location of these overexpressed receptors after acute nicotine treatment, considering that they might be due to an upregulation on either presynaptic GABA/GLU input, glial cells or LHb cell dendrites.

Despite the increase in 5-HT_2A_R-IR, we did not observe any change in the electrophysiological responses of the LHb neurons induced by systemic administration of TCB-2 in acute nicotine-treated animals compared to those reported in drug naïve animals, with the majority (52%) of the LHb neurons being decreased of about 60% of their firing rate, and the 22% of the neurons being excited (~+110%) by TCB-2. Surprisingly, TCB-2 administration in chronic nicotine-treated animals did not cause any significant excitation, as seen in acute-treated (present results) and drug-naïve animals [34], while it was still capable of inducing a similar neuronal inhibition. This might suggest that the changes in 5-HT_2A_R expression observed in the LHb at the level of the local neural processes or in other areas innervating the LHb were functionally balanced in acute, but not in chronic nicotine treated animals. A supersensitivity of 5-HT_1A_R response to 5-HT_1A_R agonist 8-OH-DPAT treatment was observed after nicotine withdrawal with the same nicotine dose of 6 mg/kg/day used in the current study in the DRN, but not after chronic treatment [38]. A later c-Fos study partially confirmed these findings that chronic treatment and nicotine withdrawal led to a robust 5-HT_1A_R-mediated inhibition in the caudal pole of the DRN [39]. Our electrophysiological evidence instead shows, for the first time, the possibility of a change in electrophysiological response pattern to a 5-HTR stimulation after nicotine treatment in the LHb with the occlusion of the excitatory response after chronic nicotine treatment. Further experiments will be necessary to clarify the biological mechanism of such a shift in the 5-HT modulation of the LHb neuronal activity under chronic nicotine exposure.

Nicotine increases LHb neuron activity when acutely administered in vivo [26], depolarizing directly LHb neurons via postsynaptic α6-containing (α6*) nAChRs but also modulating GABA and GLU input onto LHb neurons [40]. The involvement of 5-HT inputs on the nicotine effect in the LHb is also probable. Moreover, 5-HT_2A/2C_Rs are functionally expressed in the LHb [34] and mediate presynaptic 5-HT-induced potentiation of GLU transmission [41], and postsynaptic LHb neuronal excitation [42] in vitro, and mixed-effects in vivo [34]. Further, 5-HT_2A_R/nicotine interaction in the LHb can be also indirect since nicotine induces the release of 5-HT in different brain areas [36,43] that project to LHb such as DRN [30,31].

Our IHC findings show that 5-HT_2A_Rs are expressed in areas related to the nicotine effect, with a cellular and subcellular distribution in drug-naïve rats in line with previous evidence [23,44]. We are the first to report bidirectional changes in 5-HT_2A_R expression after acute nicotine treatment in the LHb (increase), mPFC (decrease), DG, and VTA (increase) neuropil, and no change in the number of the neurons in the LHb, mPFC, NAc, ST, VTA, SNc, DRN. In line with our results, acute nicotine (0.4 mg/kg) after five days of repeated vehicle administration increased [^3^H]ketanserin binding to 5-HT_2A_Rs in the VTA. Only in the hippocampal DG, we observed a robust increase (more than doubled) of the 5-HT_2A_R-IR neurons. As far as the chronic nicotine effect on 5-HT_2A_R expression is concerned, we found an increase only in the neuropil expression of the NAc while repeated treatment with nicotine evoked significant increases in [^3^H]ketanserin binding to 5-HT_2A_R receptors in the mPFC, ST, and VTA [24]. The difference in the length of the chronic treatment (5 days vs. our 21 days) and dose (0.4 mg/kg day versus our 6 mg/kg/day) used might explain the different results.

Moreover, the authors did not protect the possible binding of ketanserin on the “tetrabenazine” site (monoamine vesicular transporter), which could contribute to the effects, particularly in brain regions densely innervated with monoaminergic neurons [45,46]. On the other hand, IHC is more of a qualitative approach at variance with quantitative autoradiographic studies. Based on the present data, it is challenging to sort out the precise profile of the plastic changes associated with the effect of nicotine in the CNS. The changes could be quantitative and qualitative in a few brain regions, thereby leading to an imbalance of 5-HT_2A_R transmission within the networks associated with nicotine effects. 

We previously showed that acute nicotine induces an increase of the LHb neuronal activity in vivo [42] that might be related to its noxious/aversive effect [29,47] and reduced rewarding effect by inhibiting VTA dopaminergic neurons via the rostromedial tegmental neurons [48]. Here we found that 5-HT_2A_Rs are overexpressed in the LHb after exposure to acute nicotine and TCB-2 administration induces mainly inhibitory effects on LHb neurons of acute-treated animals. Therefore, TCB-2 might be able to counteract the nicotine-induced excitation of LHb activity. This effect mediated by 5-HT_2A_R would reduce that initial aversion due to the first cigarette and may promote nicotine addiction [49]. In line with our findings, it has been shown that 5-HT_2A_R activation opposes some of the behavioral effects of nicotine. For instance, 2,5-dimethoxy-4-iodoamphetamine ((±)-DOI), a 5-HT_2A/2C_R agonist, blocked the development of the sensitization to the locomotor effects and its associated dopamine release in the NAc of repeated nicotine in rats but did not alter the acute stimulant effect of nicotine [50]. Moreover, DOI prevented the initial suppression of locomotion induced by nicotine [51] and attenuates the discriminative stimulus properties of nicotine [51,52].

On the other hand, it has been shown that 5-HT_2A_Rs mediate some of the nicotine effects. Indeed, nicotine cognitive and reinforcing effects were blocked by ketanserin [53,54], and M100907, a selective 5-HT_2A_R antagonist prevented reinstatement of nicotine self-administration by nicotine prime or drug-associated cue [20] and nicotine sensitization [55]. Here, we showed that acute nicotine enhanced 5-HT_2A_R-IR neurons of the DG, this might contribute to nicotine-enhanced hippocampal-dependent learning [56] considering 5-HT_2A_R participates significantly to the well-documented contribution of 5-HT to memory [57,58]. TCB-2, indeed, can improve cognitive dysfunction [59] and enhance hippocampal and amygdala-dependent memory [60]. The increased expression of 5-HT_2A_Rs in the neuropil of mPFC and VTA might contribute to nicotine-induced enhanced cognition [61] and positive reinforcement [18]. Our data show that during chronic nicotine exposure, TCB-2 by sharply reducing the neuronal activity of the LHb neurons might contextually decrease nicotine aversion facilitating and sustaining nicotine addiction. Moreover, the 5-HT_2A_R activity also seems to be involved in mediating physical nicotine dependence, and M100907 and pimavanserin reduced the symptoms of nicotine withdrawal syndrome [62]. In addition, nicotine withdrawal increased [^3^H]ketanserin binding to 5-HT_2A_R receptors in the VTA and ventral DG, yet decreased binding in the NAc shell [63]. 

The major limitation of our study is that nicotine was peripherally administered. Therefore, the changes observed here on the 5-HT_2A_R system after acute and chronic nicotine exposure might be different from that induced in animals allowed to voluntary nicotine intake. Moreover, we have used a high dose of nicotine that might correlate with only high blood concentration of heavy smokers. This might be biased since it is known that the nicotine effect on the LHb, for example, changes according to the dose [42]. 

In conclusion, our data are important in the context of the mechanisms involving the 5-HT_2A_Rs, the LHb and other brain areas in nicotine addiction. Nevertheless, additional data are warranted to further our understanding of the myriad influences of 5-HT_2A_Rs and untangle the brain circuitry behind the effects of TCB-2 in the LHb during nicotine exposure. Along with other laboratories, we suggest that 5-HT_2A_R agonists sustain nicotine addictive properties, while antagonists may have a role in the development of new treatment for nicotine dependence in humans. 

## 4. Materials and Methods 

### 4.1. Animals

Male Sprague-Dawley rats, obtained from Charles River Laboratories in Margate, UK, and maintained at the Department of Physiology and Biochemistry at the University of Malta, were housed at 21 ± 1 °C, with 60 ± 5% humidity, and a 12 h light/dark cycle (lights on at 7 a.m. and off at 7 p.m.). Food and water were provided ad libitum. Adult rats that weighed 270–320 g on the day of surgery or brain extraction were used. All the animals’ procedures were in accordance with the European Union (EU) Directive 2010/63/EU and the Institutional Animal Use and Care Committee (IAUCC) of the University of Malta (1556, 10 April 2017). Utmost care was taken to limit the number of rats used and their suffering.

### 4.2. Histological Procedures and Immunocytochemistry

#### Animals and Fixation

Twenty-four rats (eight naïve nicotine, eight acute nicotine and eight chronic nicotine) were used. The animals were deeply anesthetized with a mixture (4.0 mL/kg) of sodium pentobarbital (48 mg/kg) and chloral hydrate (40 mg/kg; intraperitoneally) and perfused intracardially using a peristaltic pump (flow rate 30–35 mL/min) as follows: 0.9% saline (+4 °C) for 2 min, followed by a solution of 4% paraformaldehyde in 0.1 M sodium phosphate buffer, pH 7.4 (flow rate 10 mL/min) for 30 min. The brains were removed from the skull and postfixed in the final fixative for 2 to 4 h. The brains were then cryoprotected in 30% sucrose solution in phosphate-buffered saline (PBS), pH 7.4 at +4 °C for 48 h, and cut in the coronal plane at 30 μm section thickness on a freezing sliding microtome. The sections (1 in 5 series) were stored in 30% sucrose solution in PBS at –20 °C (for immunohistochemical staining) or in 10% formalin at room temperature (for thionin staining) until processed. 

### 4.3. Immunoperoxidase Experiments

Immunoperoxidase staining was performed on the LHb. The free-floating coronal sections were washed three times (10 min each time) in 0.02 M PBS, pH 7.4. To eliminate endogenous peroxidase activity, the sections were treated with 1% H_2_O_2_ in H_2_O for 15–30 min and then rinsed six times in 0.02 PBS. To block non-specific binding, the sections were incubated in a solution containing 10% normal goat serum (Colorado Serum Co., Denver, CO, USA, #CS 0922) and 0.3% Triton X-100 in 0.02 M PBS for 2 h at room temperature. After that, the sections were incubated in a solution containing rabbit anti-5-HT_2A_R polyclonal antibody (diluted 1:300; code 24288; ImmunoStar, Hudson, WI, USA), 0.3% Triton X-100, and 1% normal goat serum for 48 h at 4 °C. Following incubation in the primary antiserum, the sections were washed three times (10 min each) in 0.02M PBS containing 2% normal goat serum. Successively, the sections were incubated in a solution containing goat biotinylated anti-rabbit (1:200, Vector, Burlingame, CA, USA, BA-1000), 1% normal goat serum and 0.3% Triton X-100 in 0.02M PBS, pH 7.4 for 60 min at room temperature. The sections were washed three times for 10 min each in 0.02M PBS containing 2% normal goat serum and were then transferred to avidin-biotin complex (ABC kit Vectastain, PK-6100, Vector Laboratories, Burlingame, CA, USA) for 45 min. The immunoperoxidase reaction was developed by 3.3’-diaminobenzidine (DAB kit, SK-4100, Vector Laboratories, Burlingame, CA, USA). After washing, the sections were mounted onto gelatin-coated slides, dried overnight at 37 °C, defatted and intensified, according to a previous study [64], with OsO4 (0.005%, Electron Microscopy Sciences, #19130, Ft. Washington, PA, USA) and thiocarbohydrazide (0.05%, Electron Microscopy Sciences, #21900), and coverslipped with Entellan (Merck, Darmstadt, Germany).

### 4.4. Double Immunofluorescence Experiments

Double immunostaining was performed on the LHb, NAc, VTA, DRN, DG, SNc, ST, and mPFC. Preliminary experiments were carried out to determine the optimal fixative mixture to be used in the double immunofluorescence methods. The final concentrations of primary antibodies were established by performing double immunofluorescence experiments using different dilution patterns. Whole, free-floating coronal sections were washed three times (10 min each) in PBS. To block non-specific binding, the sections were incubated in 10% normal goat serum (Colorado Serum Co., Denver, CO, USA, #CS 0922) and 0.3% Triton X-100 in 0.02 M PBS for 40 min at room temperature. After that, the sections were washed three times (10 min each) in a solution containing 1% normal goat serum and 0.3% Triton X-100 in 0.02 M PBS. For colocalization studies, the sections were incubated for 2 days at 4 °C in a mixture of primary antibodies: (a) rabbit anti-5-HT_2A_R polyclonal antibody (diluted 1:300; code 24288; ImmunoStar, WI, USA) together with mouse anti-HuC/D monoclonal antibody (diluted 1:200; code A21271; Molecular Probes, Leiden, the Netherlands), which were dissolved in 1% normal goat serum (Colorado Serum Co., Denver, CO, USA, #CS 0922) and 0.3% Triton X-100 in 0.02 M PBS. The sections were washed three times as described before and incubated overnight in a secondary antibody solution containing Alexa 488-conjugated goat anti-mouse IgG (1:400, #A11029, Molecular Probes, Leiden, the Netherlands), Alexa 594-conjugated goat anti-rabbit IgG (1:400, #A11012, Molecular Probes, Leiden, the Netherlands), 1% normal goat serum, and 0.3% Triton X-100 in 0.02 M PBS. The sections were then washed with 0.02 M PBS and mounted on glass microscope slides (Super Frost Plus, Menzel-Glaser #J1800AMNZ), dried, and coverslipped using Vectashield Mounting Medium (#H-1000, Vector Laboratories, Burlingame, CA, USA).

### 4.5. Specificity of Antibodies

Rabbit polyclonal antibody anti-5-HT_2A_R is directed against an N-terminal synthetic sequence corresponding to amino acids 22–41 of rat 5-HT_2A_R. The specificity of this antibody was determined in pre-adsorption tests and Western blot studies conducted by the manufacturer and in immunohistochemical studies [65,66]. In the present experiments, control sections incubated without the primary antibody resulted in a complete disappearance of stained profiles. The specificity of mouse anti-HuC/D monoclonal antibody (code A21271; Molecular Probes) has previously been confirmed by blocking antibody-binding with the specific peptide [67]. The omission, as well as the replacement of the secondary antibodies with inappropriate secondary antibodies, resulted in the elimination of all immunohistochemical staining.

### 4.6. Thionin Staining

To help identify the boundaries of the LHb, NAcc, VTA, DRN, DG, SNp, ST, and mPFC sections adjacent to immunolabelled sections were stained with thionin as follows. Sections were taken out of the 10% formaldehyde solution, mounted on gelatin-coated slides and dried overnight at 37 °C. Sections were defatted 1 h in a mixture of chloroform/ethanol 100% (1:1), and then rehydrated through a graded series of ethanol, 2×2 min in 100% ethanol, 2 min in 96% ethanol, 2 min in 70% ethanol, 2 min in 50% ethanol, 2 min in dH_2_O, and stained 30 s in a 0.125% thionin (Fisher Scientific, Loughborough, UK) solution, dehydrated, and coverslipped with Entellan (Merck, Darmstadt, Germany).

### 4.7. Analysis of Sections

#### 4.7.1. Immunoperoxidase Experiments

Sections were analyzed using a Leica DMRB microscope. Brightfield images were acquired using a Polaroid DMC digital camera (Polaroid Corporation, Cambridge, MA, USA) and DMC 2 software. Contrast and brightness were adjusted to reflect the appearance of the labeling seen through the microscope using Adobe Photoshop CS3 Extended 10.0 software (Adobe Systems, San Jose, CA, USA).

To calculate the density of 5-HT_2A_R-IR neurons, immunostained somata were plotted bilaterally in every fifth section throughout the LHb with a computer-aided digitizing system (Accustage 5.1, St. Shoreview, MN, USA). The boundaries of the LHb were drawn from the adjacent thionin-stained sections using a stereomicroscope equipped with a drawing tube. The outlines were superimposed on computer generating plots using Corel Draw X3 (Corel Corporation, Ottawa, Ontario, Canada). The area measurements were done from the line drawings by using AxioVision Rel.4.8 (Zeiss). The density of immunostained neurons was calculated as the number of somata/mm^2^ in each section separately. For each rat, the left and right LHb located in 5 sections were analyzed. The neuronal counts are expressed as the mean number of somata/mm^2^ ± standard deviation and the data from the nicotine-naïve, acute nicotine, chronic nicotine were compared. 

#### 4.7.2. Double Immunofluorescence Experiments

Double immunofluorescence sections were analyzed with a Nikon H550L (Nikon Instruments, Tokyo, Japan) equipped with the appropriate filter cubes for immunofluorescence. We used the FITC filter for Alexa 488 (Ex 465–495; DM 505; BA 515–555) and the TRITC filter for Alexa 594 (EX 540/25; DM 565; BA 605–655). For immunofluorescence analysis, the neurons were first located by the presence of a fluorophore, which labeled one antigen, and the filter was then switched to a fluorophore specific for a different wavelength to determine whether or not the neuron was labeled for a second antigen. In this way, the proportions of neurons labeled for pairs of antigens or a single antigen were determined. Bilateral colocalization studies were assessed at the various rostrocaudal levels of the LHb. In particular, counts of single- and double-labeled neurons were carried out in five non-consecutive sections obtained from the right and left LHb, NAc, VTA, DRN, DG, SNc, ST, and mPFC of each rat. Data concerning the percentage of the image covered by 5-HT_2A_R immunoreactivity were obtained using the automatic threshold algorithm of ImageJ (version IJ 1.46r downloaded from http://imagej.nih.gov/ij/download.html). For this analysis, images were taken using a Nikon H550L (Nikon Instruments, Tokyo, Japan) microscope under identical acquisition parameters from naïve nicotine, acute nicotine and chronic nicotine. For each animal, five sections were analyzed. The images were recorded using a Nikon-Qi1Mc photo camera (Nikon Instruments, Japan) and Nikon Elements Version 4.10 software. The contrast and brightness of the figures were adjusted to reflect the appearance of the labeling seen through the microscope using Adobe Photoshop CS3 Extended 10.0 software (Adobe Systems, San Jose, CA, USA).

### 4.8. Electrophysiological Recordings

#### 4.8.1. Extracellular Single-Unit Recordings

Rats were anesthetized with chloral hydrate (400 mg/kg, i.p., Sigma-Aldrich, UK) and anesthesia was maintained with a continuous intravenous (i.v.) infusion of chloral hydrate (8% *w*/*v*; 8 mL/h). Standard single-unit extracellular recording of LHb neurons was performed as described previously [34]. Micropipettes (4–7 MΩ resistance) were positioned in the LHb (set at a 10° angle, 3.4–3.8 mm anteroposterior from bregma, 1.4–1.8 mm mediolateral from the midline, 4–5 mm dorsoventral from the cortical surface) [68]. Signal acquisition was made with a micro1401 CED laboratory interface connected to Spike2 v7.4 (Cambridge Electronic Design, Cambridge, UK) and a Neurolog amplifier and filtering system (Digitimer Ltd. UK; 10k amplification, bandpass filter set at 0.5–5 kHz). The location of the recording site was confirmed with histological analysis. 

#### 4.8.2. Drugs and Pharmacological Treatments

TCB-2 ((4-Bromo-3,6-dimethoxybenzocyclobuten-1-yl)methylamine hydrobromide) and nicotine ((-)-Nicotine hydrogen tartrate salt, (−)-1-Methyl-2-(3-pyridyl) pyrrolidine (+)-bitartrate salt) were purchased from Tocris Biosciences, UK, and Sigma Aldrich, St. Louis, MO, USA, respectively and dissolved as previously described [29,34,69]. The doses have been chosen based on previous experiments reporting their efficacy and selectivity [29,32,34,69,70,71,72]. All laboratory reagents were purchased from Sigma-Aldrich, Gillingham, UK. 

Rats from acute or chronic nicotine-treated (6 mg/kg/day for fourteen days) groups were treated with a challenge dose of 2 mg/kg, i.p. of nicotine or its vehicle (saline) one h before the recording of neurons. Once a stable neuron was detected using the procedure previously described, the rat was given i.v. through the lateral tail vein 640 µg/kg cumulative doses of TCB-2 or its vehicle. A total of eight doses (5, 5, 10, 20, 40, 80, 160 and 320 μg/kg), each dissolved in 100 μL of the vehicle were given at two-minute intervals. 

The average firing rate (FR _baseline_) from the initial 10 min baseline recording was used to normalize the data for subsequent time points (FR_x_), i.e., the 2 min following each of the eight cumulative agonist doses. Firing activity was expressed as the percentage change in firing for each time point with the baseline firing: $\left(\frac{FR_{x}-FR_{baseline}}{FR_{baseline}}\right)\times 100$. If two-time points recorded a 20% increase or a 20% decrease in neuronal activity, or none of the above, the LHb neuron was classified belonging to the increase, decrease, or null group, respectively, according to published criteria [34]. 

### 4.9. Statistical Analysis

The data report the mean ± SEM for each group if any. One-way ANOVA with repeated measures was performed separately for each treatment group compared to their respective controls. It was followed by Tukey post-hoc test (if required). A significant difference was considered when *p* < 0.05.

## Figures and Tables

**Table 1:** 5-HT_2A_R-IR somata and colocalization of HuC/D with 5-HT_2A_R in the rat lateral habenula.

Lateral Habenula (LHb)	Naïve Nicotine (NN)	Acute Nicotine (AN)	Chronic Nicotine (CN)
Density 5-HT_2A_R-IR neurons	57.1 ± 12.3	52.2 ± 13.3	51.4 ± 13.9
HuC/D-IR neurons	600	608	664
HuC/D/5-HT_2A_R-IR	64	56	56
% of 5-HT_2A_R-IR	9.6% (64/664)	8.4% (56/664)	7.8% (56/720)
% area covered by 5-HT_2A_R-IR	53.5 ± 8.8	67.7 ± 15.8 *	58.4 ± 11.6

The density of 5-HT_2A_Rs-immunoreactive somata is expressed as the mean/mm^2^ ± standard deviation. * *p* < 0.05, AN versus NN.

**Table 2:** Colocalization of HuC/D with 5-HT_2A_ receptor (5-HT_2A_R).

Medial Prefrontal Cortex (mPFC)	Naïve Nicotine (NN)	Acute Nicotine (AN)	Chronic Nicotine (CN)
HuC/D-IR neurons	242	189	317
HuC/D/5-HT_2A_R-IR	454	379	443
% of 5-HT_2A_R-IR	65.2% (454/696)	66.7% (379/568)	58.3% (443/760)
% area covered by 5-HT_2A_R-IR	75.9 ± 14.1	63.3 ± 14.1 *	76.1 ± 13.4 ^
**Dentate Gyrus (DG)**	**NN**	**AN**	**CN**
HuC/D-IR neurons	192	138	172
HuC/D/5-HT_2A_R-IR	44	99	36
% of 5-HT_2A_R-IR	18.6% (44/236)	41.8% (99/237) *	17.3% (36/208)
% area covered by 5-HT2AR-IR	57.6 ± 12.9	69.6 ± 19.7	52.1 ± 15.8 ^
**Nucleus Accumbens (NAc)**	**NN**	**AN**	**CN**
HuC/D-IR neurons	33	31	36
HuC/D/5-HT_2A_R-IR	59	49	52
% of 5-HT_2A_R-IR	64.1% (59/92)	61.3% (49/80)	59.1% (52/88)
% area covered by 5-HT_2A_R-IR	27.4 ± 8.9	33.6 ± 11.9	46.7 ± 25.4 *
**Striatum (ST)**	**NN**	**AN**	**CN**
HuC/D-IR neurons	298	302	256
HuC/D/5-HT_2A_R-IR	302	319	296
% of 5-HT_2A_R-IR	50.3% (302/600)	51.4% (319/621)	53.6% (296/552)
% area covered by 5-HT_2A_R-IR	71.8 ± 13.5	77.9 ± 14.1	67.1 ± 12.4 ^
**Ventral Tegmental Area (VTA)**	**NN**	**AN**	**CN**
HuC/D-IR neurons	88	97	104
HuC/D/5-HT_2A_R-IR	24	47	32
% of 5-HT_2A_R-IR	21.4% (24/112)	32.6% (47/144)	23.5% (32/136)
% area covered by 5-HT_2A_R-IR	45.4 ± 12.2	59.2 ± 14.9 *	54.3 ± 16.2
**Substantia Nigra Pars Compacta (SNc)**	**NN**	**AN**	**CN**
HuC/D-IR neurons	184	205	232
HuC/D/5-HT_2A_R-IR	112	91	108
% of 5-HT_2A_R-IR	37.8% (112/296)	30.7% (91/296)	31.8% (108/340)
% area covered by 5-HT_2A_R-IR	42.8 ± 12.4	49.9 ± 13.1	49.1 ± 14.5
**Dorsal raphe Nucleus (DRN)**	**NN**	**AN**	**CN**
HuC/D-IR neurons	368	284	264
HuC/D/5-HT_2A_R-IR	92	104	132
% of 5-HT_2A_R-IR	20% (92/460)	26.8% (104/388)	33.3% (132/396)
% area covered by 5-HT_2A_R-IR	19.4 ± 5.2	20.1 ± 5.9	19.1 ± 4.8

The density of 5-HT_2A_ receptors-immunoreactive somata is expressed as the mean/mm^2^ ± standard deviation. * *p* < 0.05, acute nicotine (AN) versus naïve nicotine (NN). ^ *p* < 0.05, chronic nicotine (AN) versus acute nicotine (NN).

## Supplementary Materials

Supplementary materials can be found at https://www.mdpi.com/1422-0067/21/5/1873/s1.

## Abbreviations

(±)-DOI	2,5-dimethoxy-4-iodoamphetamine
5-HT	5-hydroxytryptamine
5-HT_2C_Rs	5-HT_2C_ receptors
5-HT_A_Rs	5-HT_A_ receptors
AN	Acute nicotine
CN	Chronic nicotine
DA	Dopamine
DG	dentate gyrus
DRN	dorsal raphe nucleus
*GABA*	*Gamma-*aminobutyric acid
GLU	Glutamate
*HT2A*	5-HT_A_ receptor gene
IHC	immunohistochemistry
mPFC	Medial prefrontal cortex
NAc	nucleus accumbens
nAChRs	Nicotinic cholinergic receptors
NN	Naïve nicotine
PBS	phosphate-buffered saline
SNc	substantia nigra pars compacta
ST	Striatum
TCB-2	(4-Bromo-3,6-dimethoxybenzocyclobuten-1-yl)methylamine hydrobromide
VTA	Ventral tegmental area
