# Supplementary material for: Acute and Chronic Nicotine Exposures Differentially Affect Central Serotonin 2A Receptor Function: Focus on the Lateral Habenula
Source: Int J Mol Sci. 2020 Mar 9;21(5):1873. doi: 10.3390/ijms21051873 (PMC7084359; doi:10.3390/ijms21051873)
Supplement: Supplementary file 1 [file ijms-21-01873-s001.pdf]

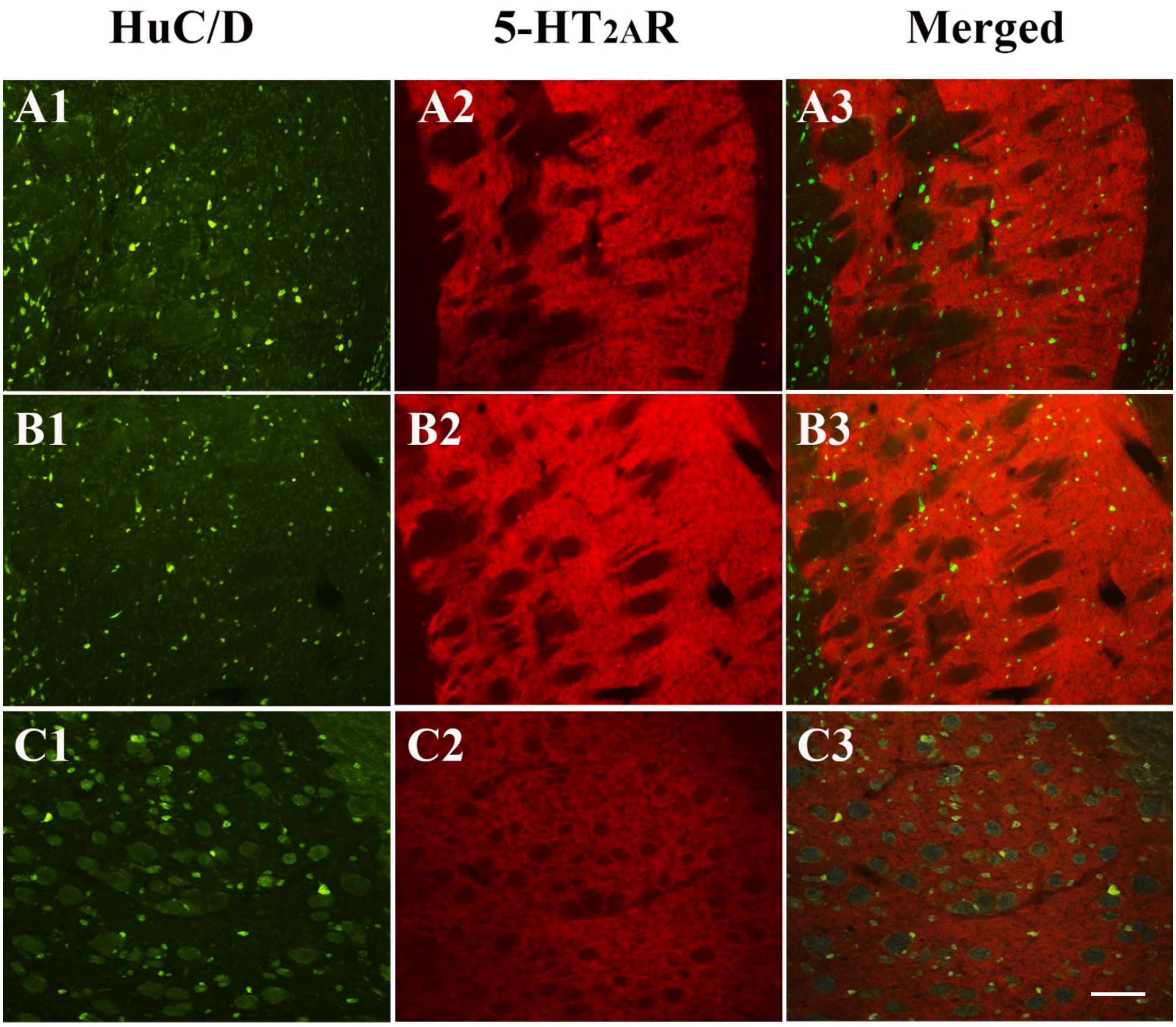

Figure 1S. Colocalization of the HuC/D with 5-HT_{2A} receptor (5-HT_{2A}R) in the striatum of drug naive (A1-A3), acute nicotine (B1-B3) and chronic nicotine (C1-C3) rats. Double immunofluorescence images showing HuC/D in green (left column pictures; A1, B1 and C1), 5-HT_{2A}R in red (middle column pictures; A2, B2 and D2), and colocalization of HuC/D with 5-HT2AR in yellow (right column merging pictures; A3, B3, C3 and D3). Note the high 5-HT_{2A} receptor immunoreactivity in the striatum of acute nicotine rats. See text and table 8 for explanations. Scale bar = 200 μm in D3 (applies to A1-C3).

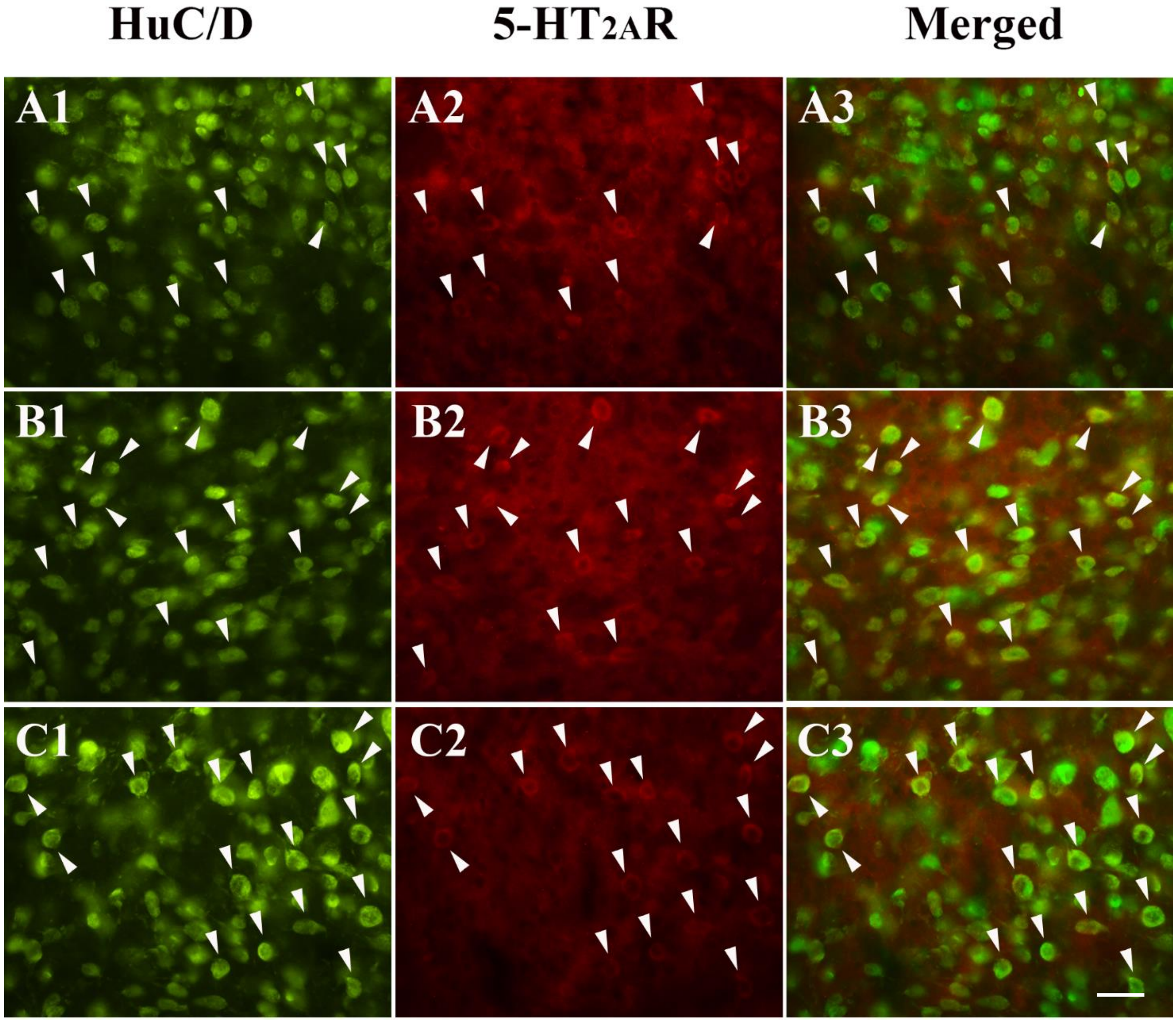

Figure 2S. Colocalization of the HuC/D with 5-HT$_{2A}$ receptor (5-HT$_{2A}$R) in the striatum of drug naive (A1-A3), acute nicotine (B1-B3) and chronic nicotine (C1-C3) rats. Double immunofluorescence images showing HuC/D in green (left column pictures; A1, B1, C1, and D1), 5-HT2AR in red (middle column pictures; A2, B2 and C2), and colocalization of HuC/D with 5-HT2AR in yellow (right column merging pictures; A3, B3 and C3 arrowheads indicate double-labeled neurons). The proportion of 5-HT$_{2A}$R-immunoreactive neurons to the total neurons does not show significant differences comparing the different groups. See text and table 8 for explanations. Scale bar = 25 μm in D3 (applies to A1-C3).

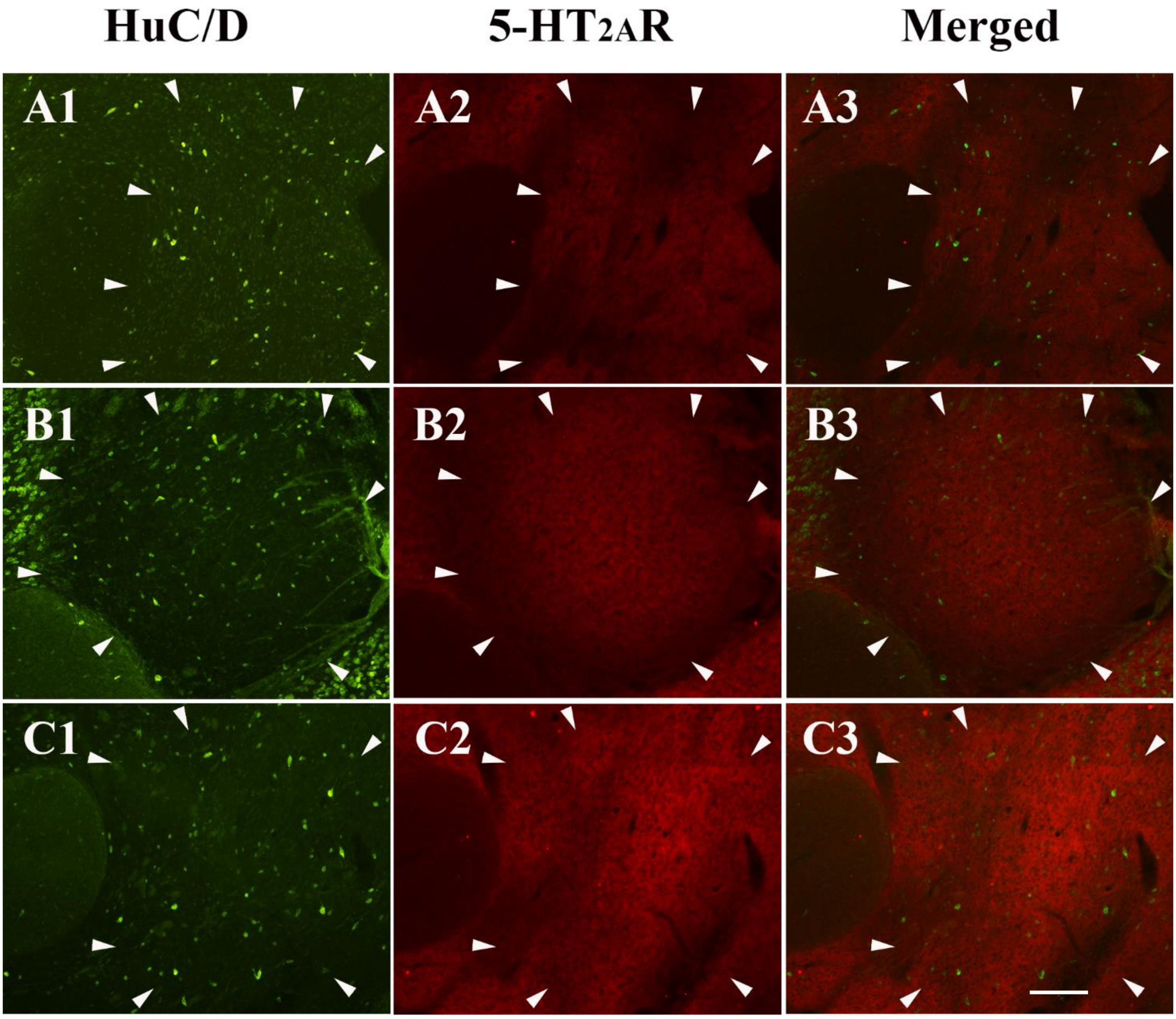

Figure 3S. Colocalization of the HuC/D with 5-HT2A receptor (5-HT2AR) in the nucleus accumbens (bordered by arrowheads) of drug naive (A1-A3), acute nicotine (B1-B3) and chronic nicotine (C1-C3 rats. Double immunofluorescence images showing HuC/D in green (left column pictures; A1, B1 and C1), 5-HT2AR in red (middle column pictures; A2, B2 and C2), and colocalization of HuC/D with 5-HT2AR in yellow (right column merging pictures; A3, B3 and C3). Note the high 5-HT_{2A} receptor immunoreactivity in the nucleus accumbens of chronic nicotine rats. See text and table 3 for explanations. Scale bar = 200 μm in C3 (applies to A1-C3).

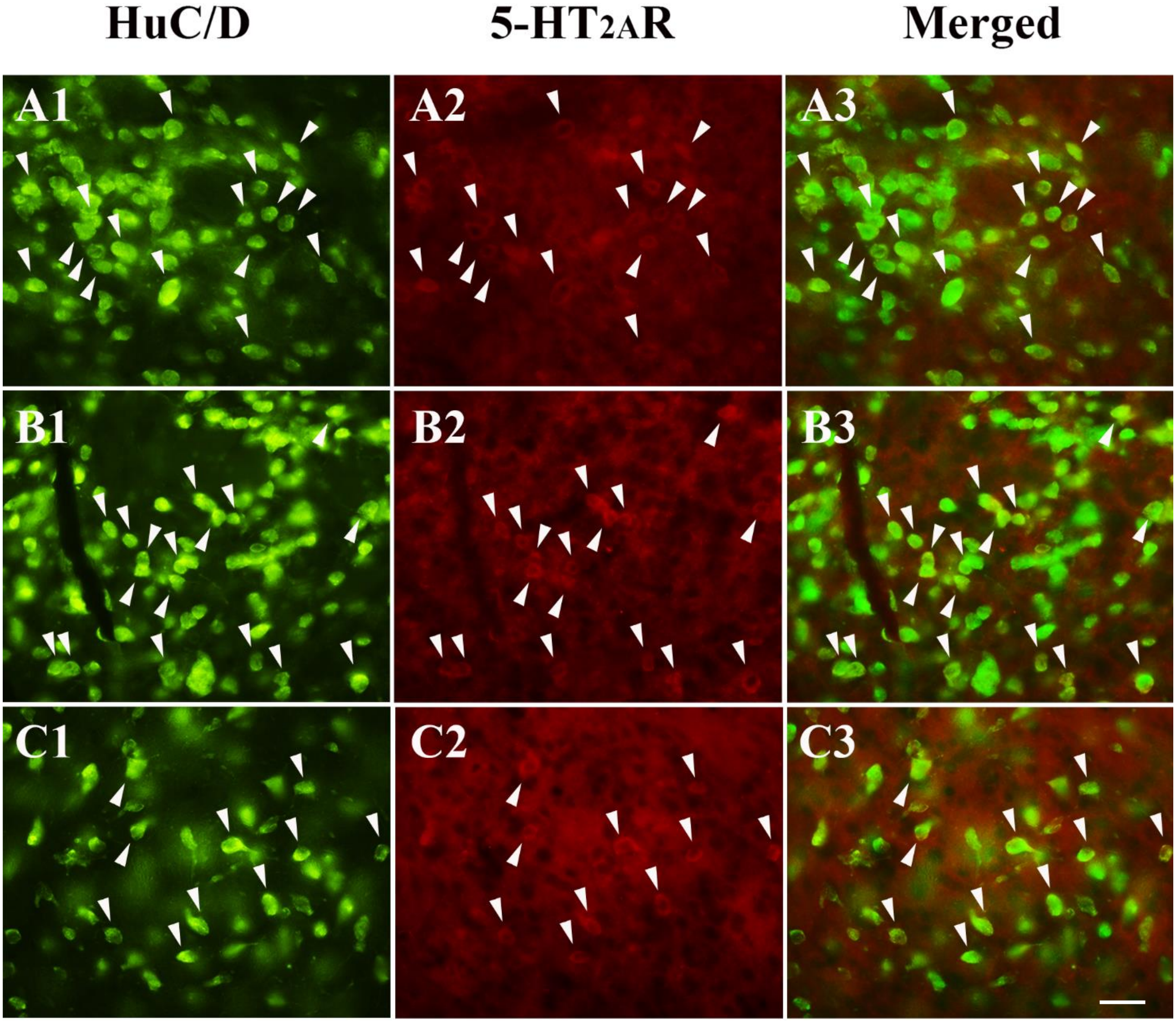

Figure 4S. Colocalization of the HuC/D with 5-HT2A receptor (5-HT2AR) in the nucleus accumbens of drug naive (A1-A3), acute nicotine (B1-B3) and chronic nicotine (C1-C3) rats. Double immunofluorescence images showing HuC/D in green (left column pictures; A1, B1 and C1), 5-HT2AR in red (middle column pictures; A2, B2 and C2), and colocalization of HuC/D with 5-HT2AR in yellow (right column merging pictures; A3, B3 and C3 arrowheads indicate double-labeled neurons). The proportion of 5-HT2AR-immunoreactive neurons to the total neurons does not show significant differences comparing the different groups. See text and table 3 for explanations. Scale bar = 25 μm in C3 (applies to A1-C3).

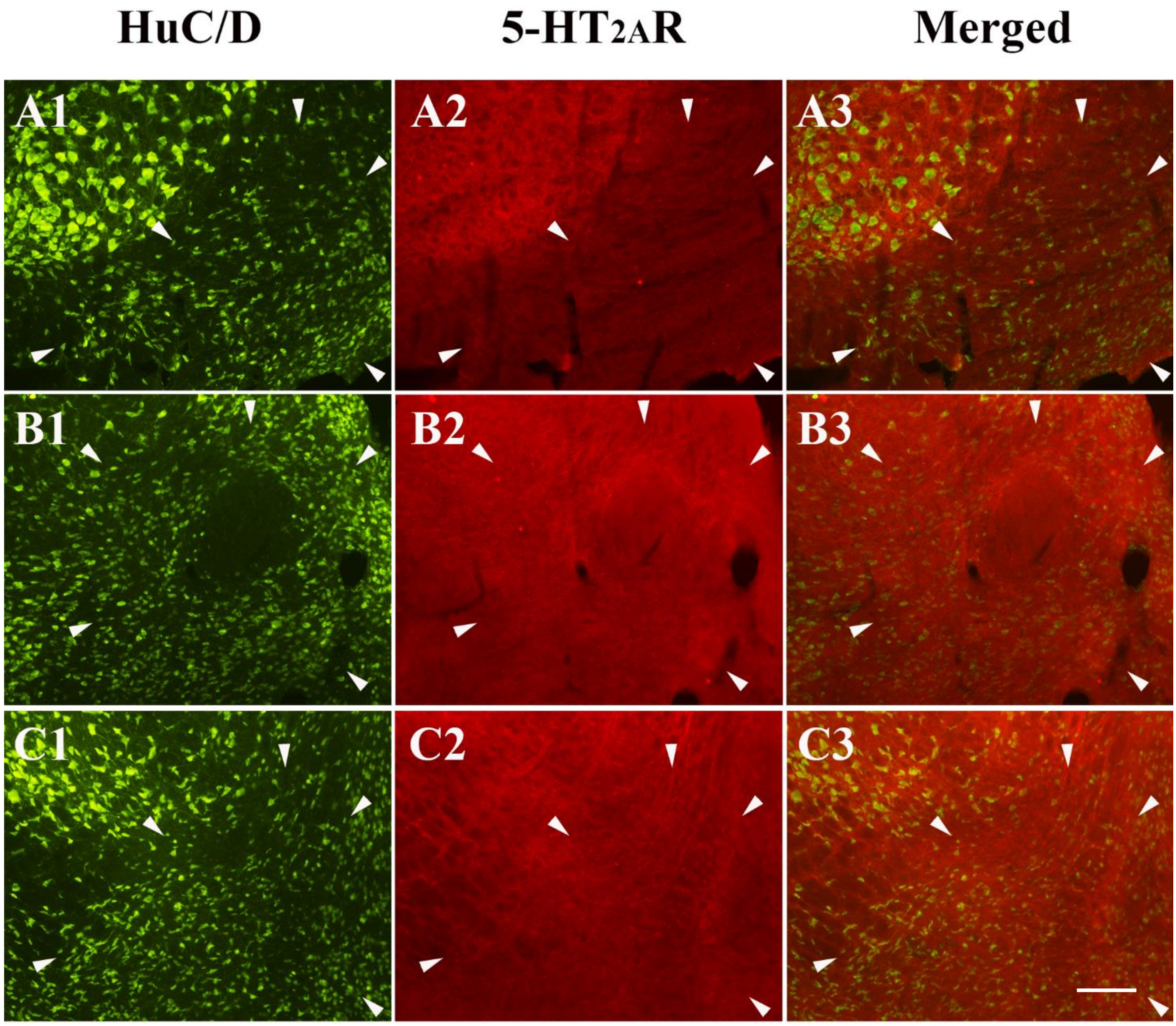

Figure 5S. Colocalization of the HuC/D with 5-HT2A receptor (5-HT2AR) in the ventral tegmental area (bordered by arrowheads) of drug-naive (A1-A3), acute nicotine (B1-B3) and chronic nicotine (C1-C3) rats. Double immunofluorescence images showing HuC/D in green (left column pictures; A1, B1 and C1), 5-HT2AR in red (middle column pictures; A2, B2 and C2), and colocalization of HuC/D with 5-HT2AR in yellow (right column merging pictures; A3, B3 and C3). Note the high 5-HT_{2A} receptor immunoreactivity in the ventral tegmental area of acute nicotine and chronic nicotine withdrawal rats. See text and table 4 for explanations. Scale bar = 200 µm in C3 (applies to A1-C3).

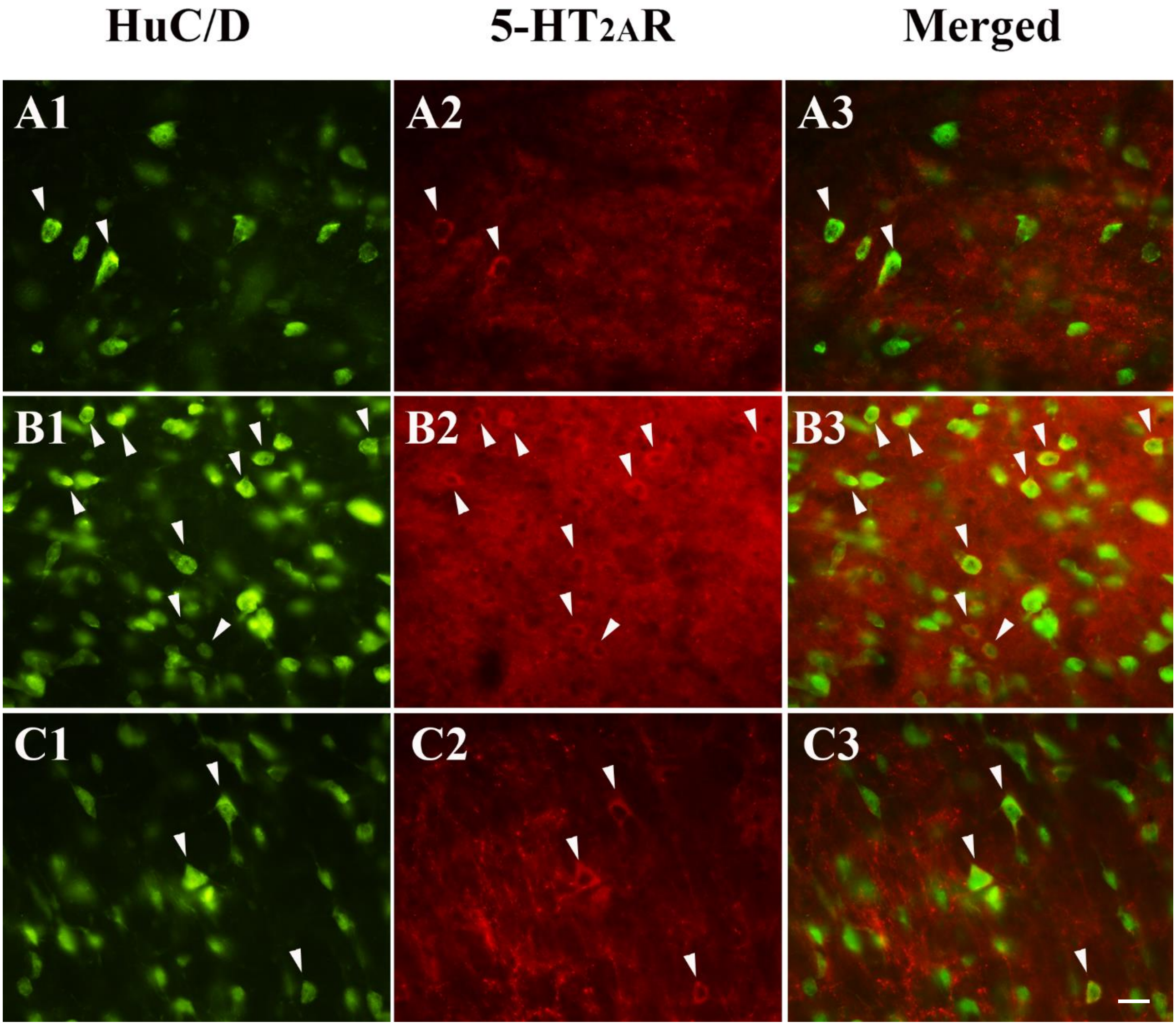

Figure 6S. Colocalization of the HuC/D with 5-HT2A receptor (5-HT2AR) in the ventral tegmental area (bordered by arrowheads) of drug-naive (A1-A3), acute nicotine (B1-B3) and chronic nicotine (C1-C3) rats. Double immunofluorescence images showing HuC/D in green (left column pictures; A1, B1 and C1), 5-HT2AR in red (middle column pictures; A2, B2 and C2), and colocalization of HuC/D with 5-HT2AR in yellow (right column merging pictures; A3, B3 and C3). The proportion of 5-HT2AR-immunoreactive neurons to the total neurons does not show significant differences comparing the different groups. See text and table 4 for explanations. Scale bar = 25 μm in C3 (applies to A1-C3).

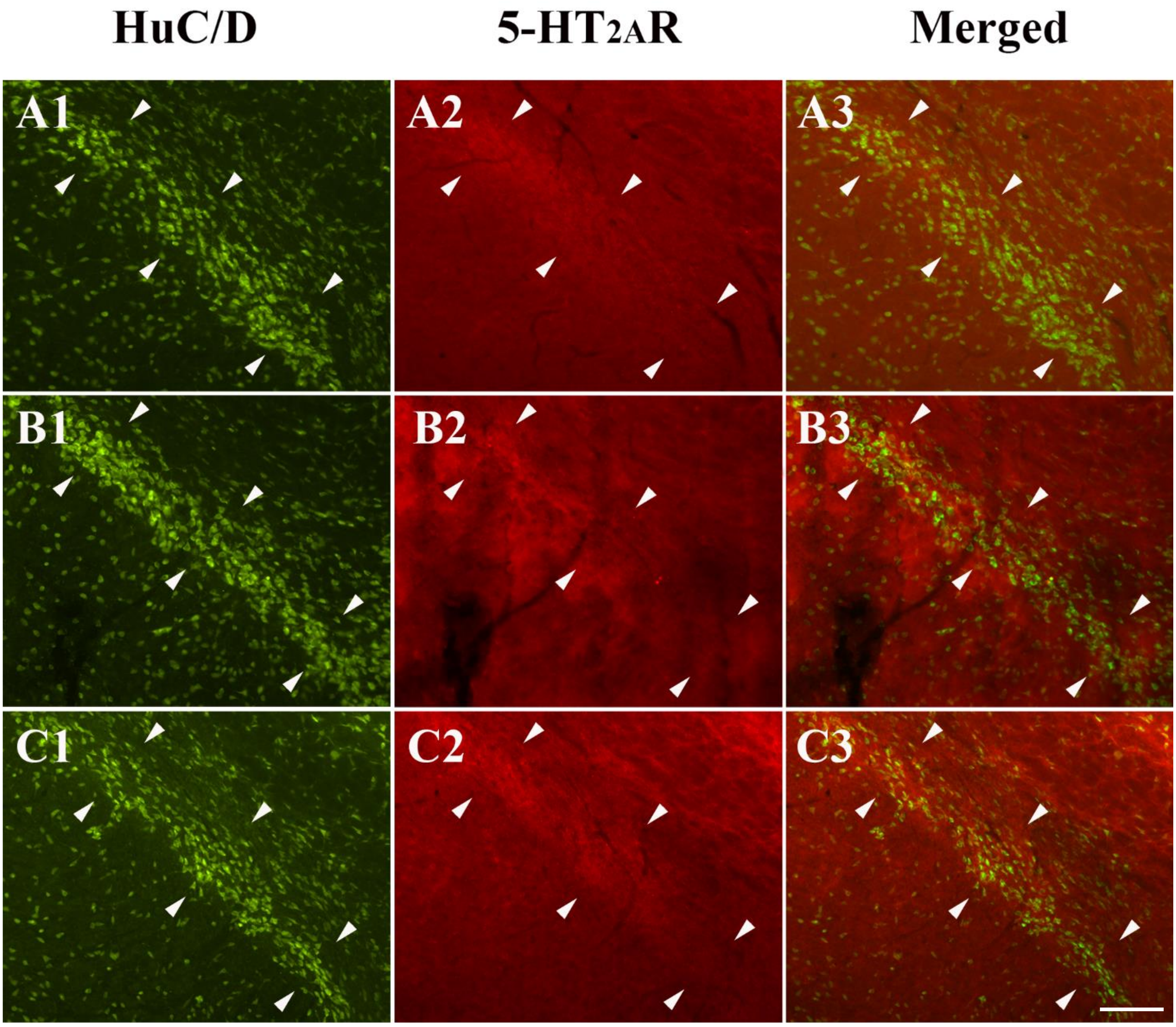

Figure 7S. Colocalization of the HuC/D with 5-HT2A receptor (5-HT2AR) in the substantia nigra pars compacta (bordered by arrowheads) of drug-naive (A1-A3), acute nicotine (B1-B3) and chronic nicotine (C1-C3) rats. Double immunofluorescence images showing HuC/D in green (left column pictures; A1, B1 and C1), 5-HT2AR in red (middle column pictures; A2, B2 and C2), and colocalization of HuC/D with 5-HT2AR in yellow (right column merging pictures; A3, B3, C3 and D3). The immunoreactivity for the 5-HT2AR is similar in the different groups. See the text and table 7 for explanations. Scale bar = 200 μm in C3 (applies to A1-C3).

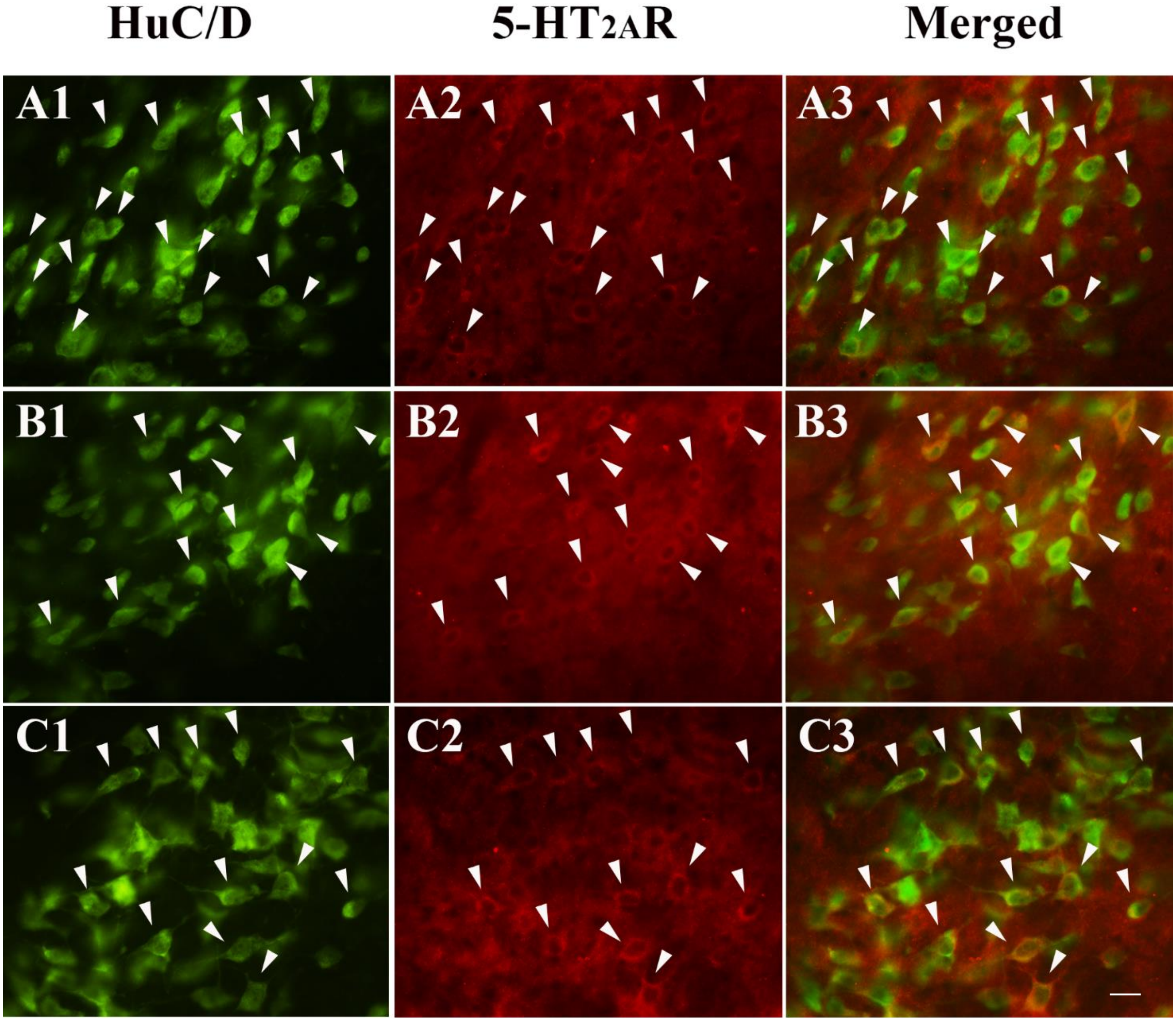

Figure 8S. Colocalization of the HuC/D with 5-HT2A receptor (5-HT2AR) in the substantia nigra pars compacta (bordered by arrowheads) of drug-naive (A1-A3), acute nicotine (B1-B3) and chronic nicotine (C1-C3) rats. Double immunofluorescence images showing HuC/D in green (left column pictures; A1, B1 and C1), 5-HT2AR in red (middle column pictures; A2, B2 and C2), and colocalization of HuC/D with 5-HT2AR in yellow (right column merging pictures; A3, B3, C3 and D3). The proportion of 5-HT2AR-immunoreactive neurons to the total neurons does not show significant differences comparing the different groups. See text and table 7 for explanations. Scale bar = 25 μm in C3 (applies to A1-C3).
